# Tracheal microenvironment, ANP metabolism and airway tone

**DOI:** 10.1007/s11434-016-1170-3

**Published:** 2016-09-22

**Authors:** Qipu Wang, Kuikui Jiang, Wanying Zhang, Wenying Qiu, Yijia Li, Yiqing Zheng, Chen Wang, Jimin Cao

**Affiliations:** 1 Department of Medicine, Peking Union Medical College, Chinese Academy of Medical Sciences, Beijing, 100032 China; 2 Department of Anatomy, Histology and Embryology, Institute of Basic Medical Sciences, Chinese Academy of Medical Sciences, School of Basic Medicine, Peking Union Medical College, Beijing, 100032 China; 3 Department of Physiology, Institute of Basic Medical Sciences, Chinese Academy of Medical Sciences, School of Basic Medicine, Peking Union Medical College, Beijing, 100032 China

Asthma is a chronic inflammatory disease, characterized by reversible airflow obstruction and airway hyperresponsiveness. There has been a sharp increase in the prevalence, morbidity, mortality and economic burden associated with this disorder over the last several decades, which triggers the development of various antiasthmatic agents [1]. Maintenance of airway tone is the key in asthma therapy. This role is currently performed by bronchodilators. β2-adrenoceptor (β2-AR) agonists are gold standard asthma therapeutics, resulting in bronchodilation via the β2-AR-cyclic adenosine monophosphate (cAMP) signaling. However, chronic application of β2-AR agonists is associated with desensitization of downstream signaling, worsening of airway hyperreactivity, and increased incidence of asthma-related clinical events [2]. Therefore, researchers are attempting to identify novel non-β-agonist bronchodilators, in which natriuretic peptides are attractive candidates.

The natriuretic peptides are hormones with important roles in water and salt homeostasis. Heart has long been thought as the only site of natriuretic peptide production. However, recent studies show that lung is another source, especially for atrial natriuretic peptide (ANP). For example, airway epithelium and smooth muscle cells have been shown to synthesize ANP, exerting only paracrine effects, and its expression can be enhanced by hypoxia [3]. Tracheal chondrocytes express both neutral endopeptidase (NEP) and natriuretic peptide receptor-C (NPR-C) [4]. Due to its unique downstream signaling and effective regulatory mechanism, ANP has been considered to be a promising candidate in novel bronchodilator development. Like many peptide hormones, ANP is secreted as a propeptide and then converted into its active form by a specific transmembrane protease, herein Corin [5]. ANP is a potent relaxant of both intrinsic and extrinsic stimuli-induced airway contraction. Through its receptor, ANP leads to cyclic guanosine monophosphate (cGMP) accumulation in the airway smooth muscles and results in bronchodilation [3]. As ANP has potent physiological effect, its activity is regulated tightly and will be diminished rapidly when it enters the circulation. Two main mechanisms contribute to this process: the clearance receptor natriuretic peptide receptor-C (NPR-C) mediated ANP endocytosis, and the extracellular peptidase (mainly neutral endopeptidase, NEP) mediated ANP degradation [6]. All these properties make ANP an ideal candidate of bronchodilators and prompt researchers to explore its efficacy in clinical studies [7].

Inhalation is widely accepted as the optimal route of medication administration for asthma. Benefits of pulmonary drug delivery include increased local therapeutic response and decreased redistribution of the inhaled agents in the circulation, which is directly related to the side effects [8]. Recent advances emphasize that the therapeutic effect of inhaled bronchodilators is not only determined by their action on airway smooth muscles, but also influenced greatly by other components of the airway microenvironment, such as the epithelium [9] and tracheal chondrocytes [10]. The airway microenvironmental regulatory molecules are expressed in tracheal chondrocytes, epithelium and tracheal smooth muscles and regulate the metabolism of ANP. Among these molecules, Corin catalyzes the pro-ANP into ANP, NPR-A is the receptor of ANP on tracheal smooth muscles, NPR-C mediates the internalization and lysosomal degradation of ANP, and NEP is an extracellular peptidase expressed in the tracheal chondrocytes and degradates ANP. The idea is that many local factors might regulate the biological activity of the bronchodilators. Moreover, our recent report also highlights the potential role of tracheal chondrocytes in the metabolism of bioactive molecules [10]. These pieces of evidence prompt us to explore the regulatory roles of tracheal components, especially chondrocytes, on the bronchodilative effect of ANP.

**Fig. 1:**
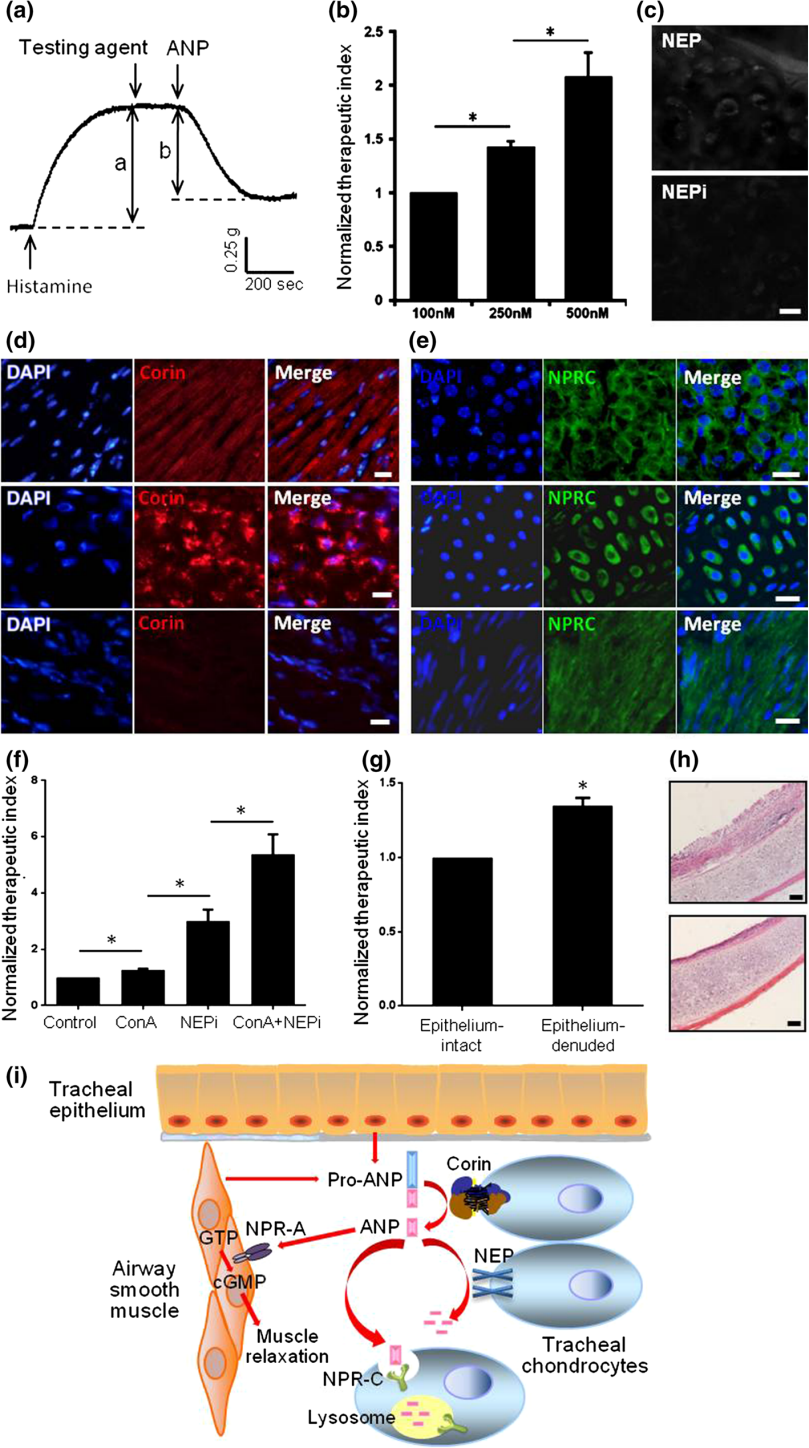
(Color online) Tracheal microenvironmental factors regulate the spasmolytic effect of atrial natriuretic peptide (ANP) in the guinea pig. **a**, **b** A schematic record of the tension curve of tracheal spirals and the calculation of therapeutic index (defined as “b/a”), and the relaxation effect of ANP at different concentrations, **c** histochemical staining of NEP on the tracheal cartilage with or without treatment of NEPi. Scale bar 20 μm, **d** immunofluorescent staining of Corin on cardiomyocytes (upper), tracheal chondrocytes (middle) and tracheal smooth muscle cells (lower). Scale bar 20 μm, **e** immunofluorescent staining of NPR-C on cells of adrenal gland cortex (upper), tracheal chondrocytes (middle) and tracheal smooth muscle cells (lower). Scale bar 20 μm, **f** pharmacological inhibition of NEP and/or NPR-C enhanced the spasmolytic effect of ANP, **g** epithelium removal resulted in stronger relaxation response to ANP, **h** microscopic appearance of the intact trachea and trachea with epithelium removal. Scale bar 100 μm. *P < 0.05, **i** schematic representation of tracheal microenvironmental factors and signaling underlying the spasmolytic effect of atrial natriuretic peptide (ANP) based on the present study and related reports. We propose trachea as a microenviroment which facilitates production, maturation, function and degradation of ANP. Firstly, tracheal epithelium and smooth muscle cells synthesize and release pro-ANP, then digested by corin, a membrane protein expressed on cartilage cell membrane, to functional ANP. By binding to NPR-A receptor, it stimulated downstream reaction and induced trachea relaxation. Meanwhile, degradation of ANP is mediated by endoproteinase NEP and cleaning receptor NPR-C

Using the histamine-sensitized guinea pig tracheal spirals, we demonstrated that besides airway smooth muscles as the direct action target, other components of the airway, including chondrocytes and epithelium, could regulate the spasmolytic action of atrial natriuretic peptide (ANP). ANP decreased the airway tone in a dose-dependent manner as indicated by the therapeutic index (Fig. 1a, b). Tracheal chondrocytes expressed NEP and was inhibited by NEPi in an enzyme activity-based histochemical method (Fig. 1c). Tracheal chondrocytes strongly expressed Corin (Fig. 1d) as compared with the positive control cardiomyocytes (Fig. 1d), but Corin was negative in tracheal smooth muscle cells (Fig. 1d). Both tracheal chondrocytes and tracheal smooth muscle cells expressed NPR-C (Fig. 1e) as compared with the positive control adrenal gland cortex (Fig. 1e). We further demonstrated that the trachea-expressed NPR-C and NEP mediated the inactivation of ANP. This result also had clinical relevance, since local inactivation of inhaled ANP would determine its therapeutic duration. Concanavalin A (ConA), an inhibitor of clathrin-mediated endocytosis, only improved the spasmolytic effect of ANP moderately (Fig. 1f), which could be attributed to the disruption of the clearance effect of NPR-C. However, application of phosphoramidon, a potent inhibitor of NEP (NEPi), dramatically increased the action of ANP (Fig. 1f). This difference indicated that NEP mediated degradation, rather than NPR-C mediated endocytosis, predominated the inactivation of local ANP in the guinea pig trachea. As expected, administration of the two inhibitory agents together could achieve a synergistic effect (Fig. 1f). In addition, we found that tracheal epithelium had significant influence on the ANP action. ANP showed enhanced spasmolytic effect on the epithelium-denuded trachea (Fig. 1g). Removal of the epithelium and the integrity of the surrounding tissues were confirmed by histology (Fig. 1h). Mechanistically, there was NEP expression in the tracheal epithelium. Thus, when the epithelium was removed, the action of ANP could be prolonged. However, this observation did not deny the presence of epithelium-transduced relaxation effect, as the guinea pig trachea spiral used in our study to measure the smooth muscle tension has already impaired the integrity of the epithelium.

Based on these findings, we outlined the signaling pathways (Fig. 1i) to depict how the airway microenvironmental components regulate the ANP action on airway tone. Tracheal epithelium and smooth muscle cells synthetize and release pro-ANP, which is cut by Corin and activated to ANP. ANP acts on its receptor NPR-A on the smooth muscle cells, thus dilating the airway. The degradation of ANP is mediated by NEP and NPR-C, which are both expressed on the tracheal chondrocytes. Overall, these observations imply that the regulatory components of ANP in the tracheal microenvironment may have great impact on the effect of bronchodilators, and could be potential medication targets in furture clinical practice.
